# The Role of Psychological Safety in Enhancing Medical Students’ Engagement in Online Synchronous Learning

**DOI:** 10.1007/s40670-023-01753-8

**Published:** 2023-02-21

**Authors:** Emma McLeod, Shalini Gupta

**Affiliations:** School of Medicine, University of Dundee, Dundee, UK

**Keywords:** Psychological safety, COVID-19, Medical education, Learning, Online teaching

## Abstract

**Background:**

The COVID-19 pandemic instigated a global change in the delivery of undergraduate medical education, with an eminent shift from in-person to online teaching. The virtual methods that were utilised to a limited extent previously have now become the mainstay in education. The concept of psychological safety has been studied previously within medical education, but not in the distance learning context. The aim of the study was to explore students’ experiences of online learning and to gain an understanding of the factors affecting psychological safety and its subsequent impact on their learning.

**Methods:**

A qualitative, social constructivist approach was adopted in this research. Data collection involved semi-structured interviews with 15 medical students from the University of Dundee. There was a representation from each year group on the undergraduate medical course. Data was transcribed verbatim and analysed thematically.

**Results:**

Five key themes were identified as motivation for learning, engagement with learning, fear of judgement, group learning and adjustment to online learning. Each of these comprised of interlinked subthemes related to peer and tutor interactions.

**Conclusions:**

Drawing on students’ experiences, the paper presents the significant interplay of group interactions and tutor attributes operating in the virtual synchronous learning environment. The relevance of psychological safety in student learning and experience, and strategies to foster it in online classrooms are discussed in the context of existing literature and proposed future developments.

## Introduction

The idea that psychological safety (PS) could exist within a team was first suggested by Edmondson in 1991 and was originally described in work contexts [1]. It has been identified as a critical factor in enabling performance and collaboration through creating a climate that mitigates interpersonal risks in professional hierarchies. High levels of PS in teams allow for increased creativity, increased collaboration, admitting mistakes, asking for help, providing feedback and identifying opportunities for improvement.

In medicine, PS has been used as a lens to study error reporting in hospital setting and its consequences in terms of patient safety [2, 3]. Large power differentials in clinical teams and pervasive hierarchies creates a psychologically unsafe environment that can discourage learners from speaking up; this has been researched in postgraduate practice environments in medicine, where residents were reluctant to report adverse events [4, 5]. Likewise, in undergraduate contexts, it is perceived that medical students typically occupy a lower status position in the vertical hierarchy owing to their relative lack of training and, as such, are likely to feel less psychologically safe when working with more senior colleagues [2]. It is foundational to build PS in medical education contexts, where learners are able to engage wholeheartedly with the learning instead of managing impressions in situations characterised by asymmetries of power.

In education, students who perceive a learning environment as psychologically safe are less likely to be preoccupied with a sense of worry and more likely to actively participate in the teaching session [6]. Hence, PS in educational contexts is also renamed as educational safety, defined as:

the subjective state of feeling freed from a sense of judgement by others such that learners can authentically and wholeheartedly concentrate on engaging in a learning task without a perceived need to self-monitor their projected image. [6, p. S32].

There has also been a suggested link between motivation and PS, with intrinsic motivation improving medical students’ learning, and factors such as the learning environment and peers in the group, impacting both motivation and PS [7]. Learners in psychologically unsafe environments are less likely to engage in learning tasks at both individual and group levels [8]. It is well established that ‘educational experiences are only as effective as students’ engagement with them’ [9, p. 251], thus reinforcing the critical relationship between student engagement, motivation and overall learning. These parameters are, however, predominantly discussed in in-person teaching and learning environment, and there is little information on the relevance of PS in online learning contexts.

Building PS in a group is known to take time as group familiarity increases, and members respond positively to displays of vulnerability [10]. Medical educators are constantly striving to enhance student learning and satisfaction through adopting diverse strategies that improve student participation and engagement in the teaching sessions. Increasingly in medical education, didactic teaching methods are being replaced by interactive styles which encourage application and retention of knowledge. In a psychologically safe environment, such strategies foster adaptive expertise and critical thinking abilities which are considered key learning outcomes in contemporary education [11]. It is worth noting that these conversations around PS and teaching styles are confined to in-person teaching, and there is dearth of research illuminating these attributes in virtual learning environments.

Advances in educational technology over the years have allowed students to participate anonymously through strategies such as audience response systems (ARS) involving handheld ‘clickers’ or web-based tools, such as Kahoot and Mentimeter [12]. The immense popularity of these anonymity ensuring measures could possibly be attributed to increase in PS in the learning environment. However, the lack of qualitative research does not render confidence to the claim. Furthermore, the few existing studies on PS originate primarily from the USA where the learning context and student demographics vary. Educationalists have called for an enhanced focus on medical learners’ perceived risks and the associated factors to establish PS in the learning environment [4].

Technology-enhanced learning has enabled development and uptake of several interactive online platforms in medical education over the past two decades. Popular learning management systems include Microsoft Teams (www.microsoft.com/en-ww/microsoft-teams/groupchat-software), Google Classroom (www.classroom.google.com) and MOODLE (www.moodle.org), which the higher education institutes select on the basis of cost-efficacy, convenience for faculty and ease of integration into their existing system. Until 2020, online teaching was often found within undergraduate medical education as part of a ‘blended learning’ approach, employing both face-to-face and virtual methods of delivery. However, in March 2020, the COVID-19 pandemic triggered an eminent shift to online learning across the globe, and our medical school was no exception. Goh and Sandars predict that the use of technology within medical education will remain predominant following the pandemic [13]. Therefore, it is imperative to investigate the role of PS in online classrooms and implement strategies that advance the same.

The authors undertook the study to enhance the understanding of PS within an online learning environment, through the exploration of medical students’ experiences of participating in online synchronous classrooms. The specific research questions were:

1. What are medical students’ experiences of learning in online classrooms?
2. What do medical students perceive to be the barriers and enablers to active participation in an online classroom?
3. How do the peer and tutor dynamics within an online classroom impact PS amongst medical students?

## Materials and Methods

Our research context was the School of Medicine at the University of Dundee (UoD), which has a 5-year long MBChB (undergraduate medical) course. The first author (EM) is a medical student at the UoD, who undertook the present study under the supervision of the second author (SG), who is a faculty in medical education at the UoD. All students on the MBChB course (approximately 900 in the entire cohort) were invited to participate in this research, as each year group had experienced the shift to online teaching, to some extent, since the beginning of the COVID-19 pandemic. A qualitative approach was taken to answer the above research questions, and a social constructivist paradigm was used to enable a deep understanding of the participants’ experiences and perceptions.

In advance of commencing the study, ethical approval was granted by the UoD’s ‘School of Medicine and Life Science Research Ethics Committee’ (SMED REC Number 20/121). An invitation email was sent through the medical school office to all students enrolled in the MBChB programme employing a convenience sampling to recruit participants ‘according to their availability and accessibility’. The initial email by the school staff was sent to approximately 900 students and summarised the research objective and plan. Eighteen students responded to this open invitation, expressing an interest in the study. These respondents were then contacted by the primary researcher with the participant information sheet, and a Microsoft Teams call was arranged to provide any clarifications regarding the study. Voluntary informed consent was obtained from each of the 15 participants who enrolled. The consent form included permission for audio-recording and transcribing, and a reassurance that the interview data would be de-identified and presented cumulatively such that no individual participant could be identified.

The interview schedule was developed with questions that explored medical students’ diverse experiences (both positive and negative) of learning in online classrooms and various enablers and barriers to their participation and engagement in these sessions. The following questions served as prompts, and the interviews were further guided by the participants’ responses:

1. Could you describe a time, or times, in an online classroom when you felt comfortable participating in the learning activity?
  - What was it that made you comfortable?
  - Can you provide details, please, for example, the group you were with, the tutor(s) and the content of the session?
  - How did this influence your learning?
2. Could you describe a time, or times, in an online classroom when you felt uncomfortable participating in the learning activity?
  - What was it that made you uncomfortable?
  - Can you provide details, please, for example, the group you were with, the tutor(s) and the content of the session?
  - How did this influence your learning?
3. How can the medical school help to make online classroom more engaging and participative?

In advance of data collection, the interview guide was piloted with a fellow researcher, which allowed it to be trialled and reworded to remove ambiguity. Interviews were conducted virtually over Microsoft Teams. Towards the end of the interview, each participant was given the opportunity to expand on any answers or to add additional points they felt may be relevant to the topic. Recordings were transcribed verbatim by the primary researcher. The transcripts were thematically analysed to identify key themes related to online participation and learning following a six-step approach described by Braun and Clark [14]. Memos maintained during the data collection and iterative analysis improved confirmability during coding, categorising and generating themes. Importantly, the primary researcher (EM) maintained a reflexive diary throughout to reflect on her position, record thoughts and feelings and maintain a ‘social and emotional distance’ from the participants and collected data, to avoid her own experiences of online classrooms and PS influencing the questions asked and data collected. Trustworthiness was further enhanced by discussing preliminary codes with the supervising researcher and revisiting sections of participant transcripts where required. Results presented below seek to illustrate patterns existing in student accounts and reveal participants’ experiences of online classrooms.

## Results

The study sample comprised of 15 students, spread across each of the 5-year groups of the undergraduate medical course. The entire MBChB student population of 900 was invited, making the response rate 1.67%. Table 1 displays the participant demographics. The sample is representative of the medical student population at the University of Dundee, with respect to age and gender. Data is sourced from 15 students who consented and shared their experiences in online semi-structured interviews (ranging from 8 to 25 min).

**Table 1:** Participant demographics

Gender	Male	6
	Female	9
	Non-binary	0
	Other	0
Year group	Year 1	4
	Year 2	2
	Year 3	4
	Year 4	3
	Year 5	2
Age (years)	≤ 17	0
	18–21	9
	22–25	6
	26–32	0
	≥ 33	0
Entry to medicine	Undergraduate	14
	Postgraduate	0
	Other	1
First language	English	14
	Other	1

**Fig. 1:**
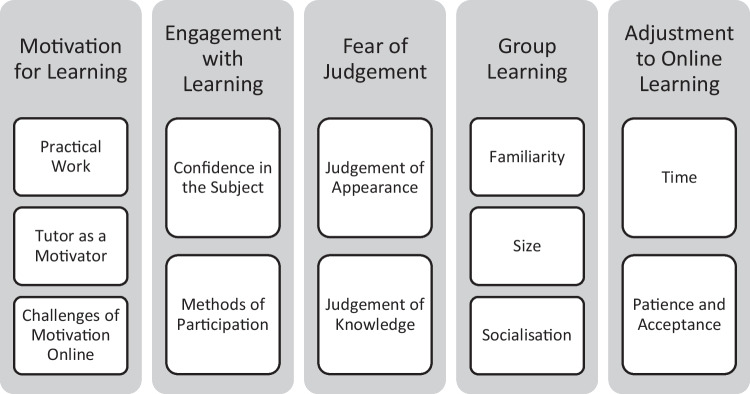
Themes and subthemes

Following transcription and analysis, five main themes were extracted from the data which included several inter-related subthemes (see Fig. 1). Themes below are supported by representative quotes from interview transcripts and also serve to highlight the natural context of the online learning environment and make the participants’ experience apparent to the readers. Within the given quotes, ellipses (…) are used to indicate where data has been condensed to increase clarity and relevance.

### Motivation for Learning

Participants reported feeling less motivated when working online. On exploration, they elaborated that the lack of face-to-face peer interactions, owing to social distancing and pandemic regulations, was the primary issue underlying reduced motivation towards course work. The cancelled timetabled clinical placements resulted in reduced levels of active participation overall:

Normally having the clinical stuff at the side would give us sort of a reminder of what medicine’s actually like and why we’re actually doing it. (Participant 5).

Participants felt that the tutor played a major role in the creation of a psychologically safe online environment. Many students reported that direct questioning adversely impacted their learning through inducing stress:

I feel like whenever the tutor’s going through calling out names it definitely influences my learning in a negative way cause I’m sitting nervous waiting for the question or thinking what I’m gonna say rather than taking in what I’m being told. (Participant 12)

On the other hand, participants also recounted incidences when the tutor attempted to make the students at ease, encouraging them to ask questions and participate. Students appreciated tutors’ extra effort to make the online classrooms less formal and more relaxed as illustrated in the following quotations:

I suppose just extra communication saying ‘please feel free to speak in this class’ and kind of facilitating a safe and comfortable environment that everyone feels happy to participate in. (Participant 6)

If they’re just kind of a bit nicer when they are asking you questions like don’t worry if you don’t know (..) I think that works quite well cause then you’re forced to think about something but again you know it’s fine even if you get it wrong. (Participant 2)

Participants also identified the challenges of online learning, namely, the increase in at-home distractions and regular unplanned interruptions in the accommodation which were unavoidable; these were felt by students to have negatively impacted their motivation:

In your own home there’s lots of things to distract you and kind of taking your focus away from learning which definitely doesn’t help. (Participant 5).

### Engagement with Learning

Similar to in-person teaching, a student’s individual confidence level in the subject were found to influence their likelihood of participation in the online class. The student below explains how their lack of confidence with reading ECGs led them to be less active during a tutorial on the subject:

For example, we had a lot of ECG tutorials and I’m not very good at ECGs so I would take a backseat during that tutorial…whereas other topics I felt more confident I’d done a bit more studying on I would kind of be answering the questions. (Participant 15).

One method of improving student engagement was identified to be the tutor’s use of anonymous options of participation, including the whiteboard function, polling features and Mentimeter:

I think that a lot more people tend to participate the minute somebody puts the whiteboard up…or even Menti. (Participant 1).

So whenever the whiteboard’s put on, you can just put on questions anonymously. I think that is what gets a lot of responses. (Participant 11).

I have asked quite a few questions on the anonymous whiteboard that I have never asked like in class, or like if I had to turn my camera on (…) you do feel self- conscious when you’re asking some question which you might feel is quite silly. (Participant 11).

### Fear of Judgement

Another key barrier prohibiting active participation in the online classroom was the participants’ fear of judgement, both on their appearance and knowledge. Many students commented on their internal debate of whether to switch their cameras on or not during the teaching sessions. Some participants identified that they may be unable to turn on their cameras due to technical issues, but many simply did not feel comfortable as they felt at risk of judgement by others.

Compared to in-person teaching, students felt that their appearance might be judged more by others in the online classrooms if they had the camera switched on, giving a feeling of being watched:

I feel like people can just stare at you more when your camera is on than like in real life, because in person you kind of look at people and then look away. (Participant 2).

However, one student identified having the camera on as positively impacting their learning, owing to the added pressure of being watched:

I’m more focussed and more aware that I’m being watched and therefore I gotta pay attention more. (Participant 9).

Additionally, concerns regarding being judged by the tutors or peers over their knowledge gaps were a constant worry with the majority of the participants, which discouraged them from voicing their queries:

You get the kind of feeling that it could be judgemental if you ask like a silly question. (Participant 7).

This concern is unrelated to the medium of instruction, and in fact, the option of using anonymous platforms (such as the whiteboard function, polls and Mentimeter as discussed in the preceding theme) in online classrooms can help overcome this.

### Group Learning

Factors influencing learning in online classrooms included group attributes such as familiarity and size and peer participation. At the UoD, medical students are assigned a ‘small group’ at the beginning of their first year, with whom the majority of tutorials and practical sessions are taught over the first 3 years of the MBChB course. Participants felt more comfortable participating in online sessions when they were in these familiar long-term groups:

When it’s my small group, probably am more likely to speak because I know sort of the people who are in my small group and I’m regularly put into breakout rooms with them. (Participant 4).

The UoD adopted BlackBoard Collaborate widely for online teaching during the pandemic, which had the ‘breakout room’ function. A larger group of students could be distributed into breakout rooms where they work as a smaller group by engaging in discussion, problem-solving and sharing content to make presentations. Participants reported having deeper interactions with a smaller group of students, as the breakout rooms elicited more participation from group members. On probing, the reasons for active participation in small group included better flow of discussion and reduced anxiety upon speaking out:

I wouldn’t be one to sort of say an answer in front of 40 people. But sort of 10 or 5 people, I’m probably a wee bit more active in participation (..) I would say a wee bit more confident in speaking up. (Participant 11).

Participants highlighted that, apart from the academic aspect of their teaching, participation in online classrooms provided opportunities for socialisation with peers. This was particularly relevant as their social lives had also been impacted by the coronavirus pandemic. Group interactions not only allowed students to strengthen the online learning community but also increased their social and cognitive presence:

Especially if most people turn their cameras on and they’re just kind of chatting (..) it definitely feels a lot more comfortable. (Participant 3).

When there’s nothing going on in-person, these small group sessions help to give you some kind of opportunity to get to know people in the year. (Participant 5).

### Adjustment to Online Learning

Tutor and student unfamiliarity with online platforms created awkward environments, which consequently decreased students’ likelihood to participate:

Sometimes it could be really awkward and stop start if they’re not very experienced with using an online platform. (Participant 15).

According to students, patience with the newly adopted online platforms and the situation was required and appreciated. Though unfamiliarity with the online platform led to participants feeling psychologically unsafe and less likely to participate, it was acknowledged that participation improved over time:

As time went on more people started being comfortable putting their cameras and their mics on (..) It was the first-time people had done online classrooms so at first people were very not willing to participate. (Participant 10).

Students identified positive experiences when their tutors were patient and accepting, citing examples of how tutors voiced their empathy and accepted their own uneasiness with online platforms to the group:

It was very comforting suddenly when the tutor said ‘This is all new to me as well and it’s new to you guys too!’ (Participant 10).

However, some students had not so positive experiences and voiced their frustrations when the tutors were more rushed, giving them inadequate time for participation:

They literally give like five seconds which it takes for people to even turn on their mic and start speaking (…) you don’t have the actual chance to even say something. (Participant 3).

Overall, a myriad of experiences related to motivation, confidence level in the subject area, group composition and time influenced students’ engagement and learning in online classrooms.

## Discussion

The present study researched medical students’ perceptions of participating in online classrooms and learning in synchronous virtual environments. The study findings confirm that some of the attributes critical for ensuring PS in in-person classrooms are valid in the online context as well, while there are certain challenges unique to the virtual climate. Smaller and familiar groups, patience and time on the part of the peers and tutors are enablers of student engagement and motivation in both virtual and in-person settings. Despite limitations and understandable barriers posed, it was universally appreciated that the online classrooms provided opportunities for peer socialisation during the pandemic and the duration of social distancing.

Several educators have emphasised that it takes time to foster PS in any group regardless of the medium of interaction and the subject matter [6, 10]. It would be reasonable to expect students’ inhibition and discomfort in the initial virtual classes, given the abrupt acceleration to online learning owing to the pandemic in March 2020. Existing research indicates that online interactions are more tiring and more isolating because of lack of access to non-verbal cues that help individuals make meaning of their social environment [15]. This results in our brains having to work overtime trying to gain this important information. In addition, the technical challenges such as lag in audio, freezing and losing connectivity are known to further exacerbate the social isolation and cognitive burden experienced [16]. Aligning with this, the participants in our study reported initial doubts and lack of confidence in the ability to achieve the relevant learning outcomes through online platforms. However, students’ familiarity and consequently their confidence and engagement grew with time. This may be attributed to a growth in PS amongst students as time elapsed, leading to improved engagement with the online tools as indicated in overlapping themes drawn from the data.

Recently, there has been much debate in medical education as well as in higher education as such, regarding the expectation from students to turn cameras on during teaching [17]. Khan et al. have highlighted the value of tutors and students having their webcams switched on to reinforce social presence in online classrooms [18]. However, the participants in our study perceived the risk of judgement to be lower when they had their cameras off. The study participants in this research appeared motivated to learn and address gaps in their knowledge. Although their reluctance and unwillingness to ask questions to avoid appearing ignorant in front of tutors and peers is suggestive of deficient PS in these online teams. In contrast, the anonymous methods of participation, such as Mentimeter, polls and the whiteboard function on Blackboard Collaborate, were hugely popular as they removed any risk of exposure or judgement related to subject knowledge. Furthermore, the use of ‘breakout rooms’ for small group collaborative learning was found to encourage participation amongst the students in this study. In our earlier letter in response to the paper by Khan et al., we reiterated the mediating role of PS in small group online learning based on the student voice in this study [19]. These findings align with existing research on the interrelationship of inherent PS amongst team members with student experience and learning in in-person setting [20]. We posit that if educators succeeded in creating psychologically safe environments, students would be encouraged to turn their cameras on without being explicitly asked.

Our research highlighted students’ struggle to maintain enthusiasm and motivation in the face of limited practical clinical exposure and usual campus experience. The study findings also confirm the presence of distractions when working from home as recognised in contemporary reviews [21]. The classroom has been traditionally defined as a physical set space in the university context. However, the pandemic triggered unplanned ‘online’ move, suddenly shifting the ‘classroom’ to bedrooms, gardens or kitchens as each participant found their own learning space. This brought its own set of challenges, as students struggled to maintain motivation for learning in the face of frequent interruptions and distractions, owing to unusual physical spaces as described by the participants in the study. Some of these barriers are beyond the control of tutors, medical school or the student themselves and so may need to be accepted as an inevitable consequence of the shift to online learning.

A significant strength of the study was that medical students from all undergraduate years were represented which allowed a capture of perceptions of a variety of online sessions. However, postgraduate representation within the cohort was missing which would have rendered potentially unique insights. Furthermore, the sample size of 15 students from a single institution who volunteered to participate in the study limits generalisability of the results. We are aware that some themes identified may be considered as artefacts of the COVID-19 pandemic and the associated rapidity of the move to online teaching and learning. It was a challenge during data analysis to separate the student experience of learning online from the experience of living through a pandemic. The researchers revisited the transcripts to better understand the context in student accounts and debated at length between themselves to minimise this confounding factor. For example, reworking with the transcripts helped us identify that the perception of online classrooms as an opportunity for peer socialisation was definitely linked to the ongoing pandemic restrictions. In a similar vein, reduced motivation towards online learning was likely related to pandemic-triggered cancellation of practical and clinical sessions. To this end, it may be safe to claim that in usual circumstances with ongoing in-person course and campus activities, students might be more motivated and engaged in online classrooms. Perhaps future research can explore and compare the online learning research from before and after the pandemic to better identify which findings are distinct from the parallel experience of living through a pandemic. We also suggest longitudinal research aiming to explore the changes in students’ PS over time as online teaching further evolves in the medical education scenario.

## Conclusions

The coronavirus pandemic has led to the widespread adoption of online learning within medical education and further afield. This study explored medical students’ experiences of online classrooms and their perceptions of the enablers and barriers to learning in such settings. This paper extends the awareness around PS amongst medical education community, given its intricate link with students’ participation, and thereby engagement and learning. The findings will be of interest to educators to foster PS in online classrooms, thus improving overall student learning and experience.

## Abbreviations

PS: Psychological safety
MBChB: Bachelor of Medicine and Bachelor of Surgery
COVID-19: novel coronavirus
